# Eg5 UFMylation promotes spindle organization during mitosis

**DOI:** 10.1038/s41419-024-06934-w

**Published:** 2024-07-31

**Authors:** Guangxu Li, Yuanjiang Huang, Wenbo Han, Liyi Wei, Hongjing Huang, Yingbao Zhu, Qiao Xiao, Zujia Wang, Wen Huang, Ranhui Duan

**Affiliations:** 1 Furong Laboratory, Center for Medical Genetics, School of Life Sciences, Central South University, Changsha, China; 2 Hunan Key Laboratory of Medical Genetics, Central South University, Changsha, China; 3 Hunan Key Laboratory of Animal Models for Human Diseases, Central South University, Changsha, China

**Keywords:** Post-translational modifications, Cell division

## Abstract

UFMylation is a highly conserved ubiquitin-like post-translational modification that catalyzes the covalent linkage of UFM1 to its target proteins. This modification plays a critical role in the maintenance of endoplasmic reticulum proteostasis, DNA damage response, autophagy, and transcriptional regulation. Mutations in *UFM1*, as well as in its specific E1 enzyme *UBA5* and E2 enzyme *UFC1*, have been genetically linked to microcephaly. Our previous research unveiled the important role of UFMylation in regulating mitosis. However, the underlying mechanisms have remained unclear due to the limited identification of substrates. In this study, we identified Eg5, a motor protein crucial for mitotic spindle assembly and maintenance, as a novel substrate for UFMylation and identified Lys564 as the crucial UFMylation site. UFMylation did not alter its transcriptional level, phosphorylation level, or protein stability, but affected the mono-ubiquitination of Eg5. During mitosis, Eg5 and UFM1 co-localize at the centrosome and spindle apparatus, and defective UFMylation leads to diminished spindle localization of Eg5. Notably, the UFMylation-defective Eg5 mutant (K564R) exhibited shorter spindles, metaphase arrest, spindle checkpoint activation, and a failure of cell division in HeLa cells. Overall, Eg5 UFMylation is essential for proper spindle organization, mitotic progression, and cell proliferation.

## Introduction

Ubiquitin-fold modifier 1 (UFM1) is a recently identified ubiquitin-like protein (UBL) that remains less well-understood compared to other UBLs [1]. UFM1 is highly conserved across evolutionary lines, with orthologs found in both Metazoa and plants [2]. Even rice shares over 66.27% nucleotide sequence similarity with humans. Although UFM1 shares only 16% amino acid sequence identity with ubiquitin, its tertiary structure closely resembles the ubiquitin fold, featuring specific β-sheets and an α-helix [3]. The UFM1 precursor consists of 85 amino acids and is processed to its mature form by specific cysteine proteases, UFSP1 and UFSP2, which cleave the C-terminal dipeptide Ser-Cys, exposing the conserved Gly83 conjugating residue [4]. The activation of UFM1 involves the UFM1-specific E1 enzyme, UBA5, through processes of adenylation and thioesterification [5]. The UFM1-specific E2 enzyme, UFC1, accepts the activated UFM1 in a trans-esterification reaction [6]. The UFM1-specific E3 enzyme, UFL1, catalyzes the covalent binding of UFM1 to substrate lysine residues [7]. The DDRGK domain-containing protein 1 (DDRGK1, also known as UFBP1) serves as an E3 ligase adapter, regulating the UFMylation of substrates [8]. UFMylation is a reversible process, as UFM1 can be cleaved from its target proteins by UFSP1 and UFSP2 [9].

UFMylation regulates various cellular functions, such as hematopoiesis, endoplasmic reticulum (ER) proteostasis, DNA damage response, autophagy, transcriptional regulation, and signaling pathways [2, 10–12]. In 2016, we reported for the first time that compound heterozygous variants of UBA5 lead to neurodevelopmental disorders characterized by cerebellar atrophy and developmental delays [13]. Subsequently, a series of biallelic variants of UBA5 were discovered in patients, causing severe infantile-onset epileptic encephalopathy primarily characterized by microcephaly [14–19]. Furthermore, biallelic mutations in UFM1 or UFC1 have revealed similar microcephalic traits [20, 21]. Genes implicated in microcephaly typically play key roles during mitosis. Our recent study in Drosophila demonstrates that knockdown of UFMylation results in reduced brain size, partial embryonic lethality before gastrulation, and mitotic defects in spindle assembly and chromosome separation [22]. However, the underlying mechanisms remain to be elucidated, due to the limited identification of relevant substrates.

Here, we employed mass spectrometry and cross-referenced other reliable proteomic datasets to identify various substrates, among which Kinesin-5 (Eg5, also referred to as KIF11) is included. Functionally, Eg5 acts as a microtubule-oriented motor protein that moves towards the plus-end, facilitating bipolar spindle assembly during mitosis [23]. Inhibition of Eg5 leads to a monopolar spindle, causing cell mitotic arrest and subsequent cell death [24].

In this study, we confirmed Eg5 as a novel substrate for UFMylation and pinpointed Lys564 as the crucial UFMylation site among its 90 lysine residues. Eg5 directly interacted with the E3 ligases UFL1 and DDRGK1, and exhibited modification by UFM1 both in vivo and in vitro. The Eg5 UFMylation predominantly occurred during mitosis, with Eg5 and UFM1 co-localizing at the centrosomes and spindle. During metaphase, defective UFMylation led to reduced spindle localization of Eg5. The UFMylation-defective Eg5 mutant (K564R) displayed shortened spindles, and cell cycle arrest at metaphase, accompanied by the activation of the spindle assembly checkpoint (SAC), culminating in the failure of the mitotic process and adversely affecting cell proliferation. Our findings suggest that Eg5 UFMylation is significant for understanding the pathogenic mechanisms involving cell cycle dysregulation.

## Materials and methods

### Cell culture

HEK-293T and HeLa cells were obtained from the American Type Culture Collection (Manassas, VA, USA) and cultured in DMEM (Logan, UT, USA) supplemented with 10% fetal bovine serum (Gibco, Waltham, MA, USA), at 37 °C in a 5% CO_2_ atmosphere. The cells used in this study were verified to be free from mycoplasma contamination, and the identity of HeLa cells was verified through STR profiling.

To generate *UFSP2* knockout cell lines, the guide RNA targeting *UFSP2* (5′-AGCAGTGACATAAACACC-3′) was cloned into pSpCas9(BB)-2A-Puro (PX459) V2.0 (62988, Addgene, Watertown, MA, USA). HEK293T cells were transfected with PX459 and selected with puromycin (1 μg/ml). After selecting for 5 days, cells were cloned using a limiting dilution and screened for knockout by Sanger sequencing and western blot analysis.

### siRNA and plasmid transfection

The siRNA duplexes were synthesized by GenePharma (Shanghai, China), and the corresponding sequences were as follows:

si-NC: UUCUUCGAACGUGUCACGU; si-*UBA5*: GGUUAUACAAGAAGAGGAA;

si-*UFC1*: GAAAGACAGCAAAGAUGUA; si-*UFL1*-1: GGAACUUGUUAAUAGCGGA;

si-*UFL1*-2: GAGGAGUAAUUUUUACGGA; si-*DDRGK1*-1: GAAAAUUGGAGCUAAGAAA;

si-*DDRGK1*-2: CCAUAAAUCGCAUCCAGGA; si-*Eg5*-1: CAGAUUGAUGUUUACCGAA;

si-*Eg5*-2: CUGGAUAUCCCAACAGGUA; si-*Eg5*-3: CGAUGAGUUUAGUGUGUAAAG.

*UBA5*, *UFC1*, *UFL1*, *DDRGK1*, and *UFM1* cDNAs were inserted into the pRK5-HA vector. UFM1 cDNA variants with two or three amino acids deleted in the C-terminal (UFM1-ΔC2 or UFM1ΔC3) were cloned into both the pRK5-HA vector and the pcDNA3.1-Flag vector. Eg5 cDNA was cloned into the pRK5-Flag vector. Truncations and point mutations of Eg5 were generated using the MutExpress II Fast Mutagenesis Kit V2 (C214-02, Vazyme, Nanjing, China). Bacteria expressing His-tagged UBA5, UFC1, UFL1, DDRGK1, UFM1-ΔC2, and GST-tagged Eg5 were generated using the pET-28a and pGEX-4T-1 systems, respectively. All plasmids were validated through Sanger sequencing. RNA interference was performed with Lipofectamine 2000 (Invitrogen, Carlsbad, CA, USA), and plasmid transfection was performed with Lipofectamine 3000 (Invitrogen), following the manufacturer’s instructions.

### Mass spectrometry

*UFSP2* knockout HEK293T cells were transfected with Flag-tagged UFM1-ΔC2 and Flag-tagged UFM1ΔC3, respectively. for 36 h. The cells were then lysed with NP-40 lysis buffer containing 50 mM Tris (pH 7.4), 150 mM NaCl, 1% NP-40, and 1× protease inhibitor cocktail (P8340, Sigma, St. Louis, MO, USA). Subsequently, Flag-UFM1-ΔC2 or Flag-UFM1ΔC3 was immunoprecipitated by incubation of the lysates with anti-FLAG M2 beads (M8823, Sigma) overnight at 4 °C. The protein precipitates were subjected to SDS-PAGE and mass spectrometry. Mass spectrometric analysis of peptide samples was carried out using a Q exactive mass spectrometer (Thermo Fisher, Waltham, MA, USA) at Applied Protein Technology Company (Shanghai, China).

### Western blot

Cells were lysed with RIPA lysis buffer consisting of 50 mM Tris (pH 7.4), 150 mM NaCl, 1% Triton X-100, 1% sodium deoxycholate, 0.1% SDS, and 1× protease inhibitor cocktail. Whole-cell lysates with equal protein were resolved by SDS-PAGE and subsequently transferred to a PVDF membrane (Millipore, Billerica, MA, USA). After blocking with 1× TBST (TBS with 0.1% Tween 20), supplemented with 5% nonfat milk for 1 h at room temperature, the membranes were incubated with the primary antibodies overnight at 4 °C and with the secondary antibodies for 1 h at room temperature.

Primary antibodies were as follows: anti-Flag (F1804, Sigma, 1:5000), anti-HA (C29F4, Cell Signaling, Danvers, MA, USA, 1:2000), anti-UFL1 (PA5-56501, Thermo Fisher, 1:1000), anti-DDRGK1 (21445-1-AP, Proteintech, Hubei, China, 1:2000), anti-Eg5 (23333-1-AP, Proteintech, 1:2000), anti-UBA5 (ab177478, Abcam, Cambridge, UK, 1:1000), anti-UFC1 (ab189251, Abcam, 1:1000), anti-UFM1 (15883-1-AP, Proteintech, 1:2000), anti-UFSP2 (ab185965, Abcam, 1:1000), anti-His (ab18184, Abcam, 1:5000), anti-α-tubulin (ab18251, Abcam, 1:5000), anti-pEg5-Thr926 (BD-PP0372, Biodragon, Suzhou, China, 1:1000), anti-pH3-Ser10 (9701, Cell Signaling, 1:1000), anti-Securin (db11881, Diagbio, Hangzhou, China, 1:1000), and anti-GAPDH (10494-1-AP, Proteintech, 1:500). Secondary antibodies were as follows: HRP-conjugated goat anti-mouse IgG (115-035-166, Jackson ImmunoResearch, West Grove, PA, USA, 1:10000) or HRP-conjugated goat anti-rabbit IgG (111-035-144, Jackson ImmunoResearch; 1:10,000). The protein bands were visualized using an ECL-chemiluminescent kit.

### UFMylation assays

For the in vivo UFMylation assays, cells transfected with appropriate plasmids were cultured for 36 h. To prepare cell lysates, the cells were boiled in a buffer containing 150 mM Tris (pH 8.0), 5% SDS, and 30% glycerol for 8 min. Subsequently, the lysates were diluted 20-fold with NP-40 lysis buffer and subjected to immunoprecipitation using anti-FLAG M2 beads overnight at 4 °C. The samples were then subjected to SDS-PAGE and western blot analysis.

In vitro, UFMylation assays were performed following the previously reported method. Recombinant GST-Eg5 was expressed in BL21 cells and purified using the GST-tag Protein Purification Kit (P2262, Beyotime, Jiangsu, China). Additionally, His-UBA5, His-UFC1, His-UFL1, His-DDRGK1, and His-UFM1-ΔC2 were expressed in BL21 cells and purified using the His-tag Protein Purification Kit (P2226, Beyotime). His-UFM1-ΔC2 (5 ng), His-UBA5 (5 ng), His-UFC1 (5 ng), His-UFL1 (5 ng), His-DDRGK1 (5 ng), and GST-Eg5 (10 ng) were mixed in the reaction buffer containing 5 mM ATP and 10 mM MgCl_2_, and incubated at 30 °C for 90 min. The mixtures were boiled with the addition of a loading buffer containing 5% mercaptoethanol for 5 min.

### Immunoprecipitation and in vitro binding assays

For immunoprecipitation, cells were lysed with NP-40 lysis buffer, and the lysates were then incubated with 1 µg of the indicated antibodies and 10 µl of Protein A/G Magnetic Beads (B23201, Biomake, Shanghai, China) overnight at 4 °C. For the in vitro binding assays, purified His-UFL1 and His-DDRGK1 were incubated with GST or GST-Eg5 in PBS with 0.2% NP-40 for 2 h at 4 °C, followed by pull-down with GSH-resin. The samples were subjected to SDS-PAGE and western blot analysis. Five percent of each supernatant was used as input control.

### Immunofluorescence staining

HeLa cells transfected with the indicated siRNAs or plasmids were cultured for 48 h. The cells were fixed in 4% paraformaldehyde for 30 min, washed in 0.2% PBST (PBS with 0.2% Triton X-100), and blocked in 5% normal goat serum for 1 h at room temperature. Cells were incubated overnight at 4 °C with primary antibodies: rabbit anti-UFM1 (ab109305, Abcam, 1:200), mouse anti-Eg5 (ab51976, Abcam, 1:500), rabbit anti-Eg5 (23333-1-AP Proteintech, 1:200), rabbit anti-α-tubulin (ab18251, Abcam, 1:500), or mouse anti-γ-tubulin (ab11316, Abcam, 1:500). Secondary antibodies were as follows: Alexa Fluor 488-conjugated goat anti-mouse IgG (115-545-003, Jackson ImmunoResearch, 1:200) or Cy3-conjugated goat anti-rabbit IgG (111-165-003, Jackson ImmunoResearch, 1:200). The cells were then stained with DAPI and mounted with Fluoromount mounting medium (F4680, Sigma). The image was acquired using the LSM 880 confocal microscope, with a Plan-Apochromat 63×/1.4 Oil DIC M27 objective (Zeiss, Oberkochen, Germany), and fluorescence intensities were calculated using ImageJ software.

### Time-lapse imaging

HeLa cells, following transfection with the specified siRNAs or plasmids, were co-transfected with H2B-mCherry (20972, Addgene). and pEGFP-Tub (VT1129, Youbio, Changsha, China) using Lipofectamine 3000 and cultured for 48 h. The microscope was situated in a temperature-controlled chamber, maintained at 37 °C, and supplied with CO_2_ to preserve physiological conditions. Time-lapse imaging of mitotic cells was conducted using an APOTOME.3 scanning system equipped with a Plan-Apochromat 63×/1.4 Oil DIC M27 objective (Zeiss). Time-lapse series were captured every minute, from the start of metaphase until the end of cytokinesis, or up to 90 min, with a resolution of 2464 × 2056 pixels.

### Cell viability and colony formation assays

For the cell viability assays, 5000 HeLa cells were seeded into 96-well plates and transfected with the indicated siRNAs or plasmids. After culturing for 24 h, 36 h, 48 h, and 72 h, the cells were treated with a CCK-8 solution (C0037, Beyotime) and incubated for 2 h. The absorbance values at a wavelength of 450 nm (OD450) were measured using an elx800 reader (BioTek, Winooski, VT, USA).

For the colony formation assays, HeLa cells were placed in 6-well plates at a density of 500 cells per well. Every three days, the cells were transfected with the indicated siRNAs or plasmids. After 10 days of culturing, during which the clones became visible, the cells were fixed using 4% paraformaldehyde and stained with 0.1% crystal violet. The colonies were then photographed and quantified.

### Statistical analysis

Statistical analyses were conducted blind to group allocation. The statistical software GraphPad Prism 8 was used to carry out unpaired Student’s t-test to compare two specific datasets and one-way ANOVA for multiple comparisons. The data were presented as mean ± SD of three independent replicates. The values of ^*^P < 0.05, ^**^P < 0.01, and ^***^P < 0.001 were considered to be statistically significant.

## Results

### Identification of candidate substrates for UFMylation

**Fig. 1:**
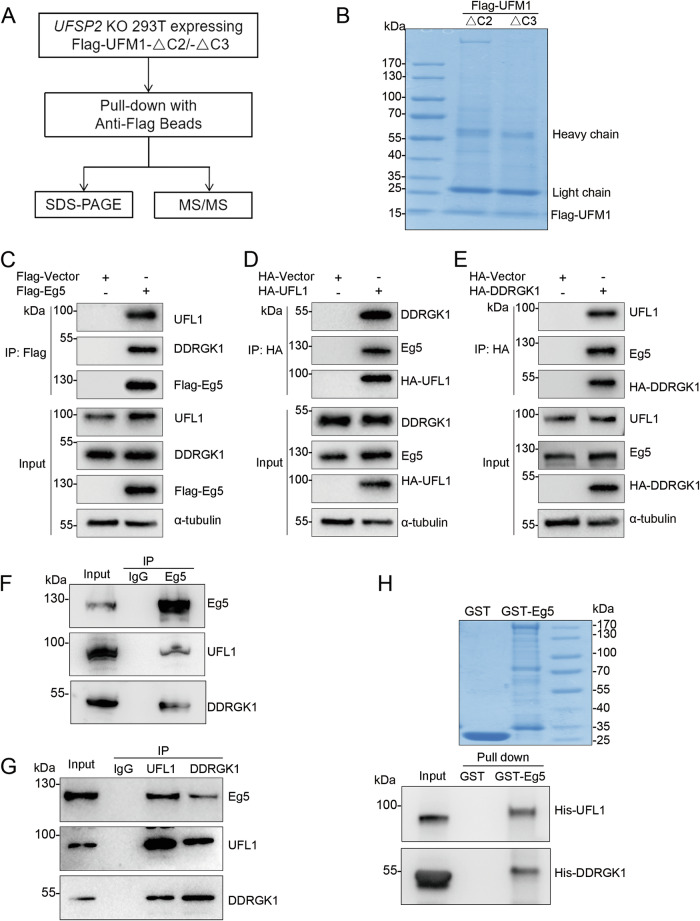
Eg5 interacts with UFL1 and DDRGK1. **A** Strategy for identification of targets for UFMylation. **B** Proteins eluted from anti-Flag M2 beads were subjected to SDS-PAGE followed by coomassie blue staining. **C**–**E** Flag-Eg5, HA-UFL1, or HA-DDRGK1 were expressed in HEK293T cells, respectively. Cell lysates were subjected to immunoprecipitation with anti-Flag or anti-HA beads followed by western blot analysis with the indicated antibodies. **F**, **G** HEK293T cell lysates were subject to immunoprecipitation with anti-Eg5, anti-UFL1, or anti-DDRGK1 antibodies followed by western blot analysis with the indicated antibodies. IgG was used as a control. **H** In vitro binding assay. Purified His-UFL1 and His-DDRGK1 were incubated with GST or GST-tagged Eg5, followed by GST pulldown assay. The samples were then subjected to western blot analysis with anti-His antibody.

To identify target proteins for UFMylation, we overexpressed flag-tagged mature UFM1 (UFM1-ΔC2) in *UFSP2* knockout HEK293T cells to facilitate UFM1 conjugate formation. In addition, we employed a Flag-tagged conjugation-defective UFM1 lacking Gly83 (UFM1-ΔC3) as a negative control (Fig. 1A). After verifying the expression levels of Flag-UFM1 (Fig. S1), we immunoprecipitated the cell lysates with anti-Flag M2 beads, and the precipitated proteins were subjected to SDS-PAGE (Fig. 1B) and mass spectrometry. We screened for UFMylation substrates by identifying proteins that specifically bound to Flag-UFM1-ΔC2 (Supplementary Table 1). Among these candidates, we selected Eg5 for further investigation due to its pivotal role in the dynamic assembly and function of the mitotic spindle through cross-linking and sliding adjacent microtubules [24].

### Eg5 interacts with UFL1 and DDRGK1

To determine whether Eg5 is a target protein for UFMylation, we examined its ability to interact with UFL1, the UFM1 E3 ligase, and DDRGK1, a critical regulatory factor for UFMylation. Immunoprecipitation analysis demonstrated that Eg5 is capable of binding to both UFL1 and DDRGK1 (Fig. 1C–E). Moreover, endogenous Eg5, UFL1, and DDRGK1 exhibited mutual interactions in cells (Fig. 1F, G). Furthermore, in vitro binding assays showed that Eg5 directly interacted with UFL1 and DDRGK 1(Fig. 1H), suggesting that Eg5 could be a bona fide substrate of UFMylation.

### Eg5 is a target substrate for UFMylation

**Fig. 2:**
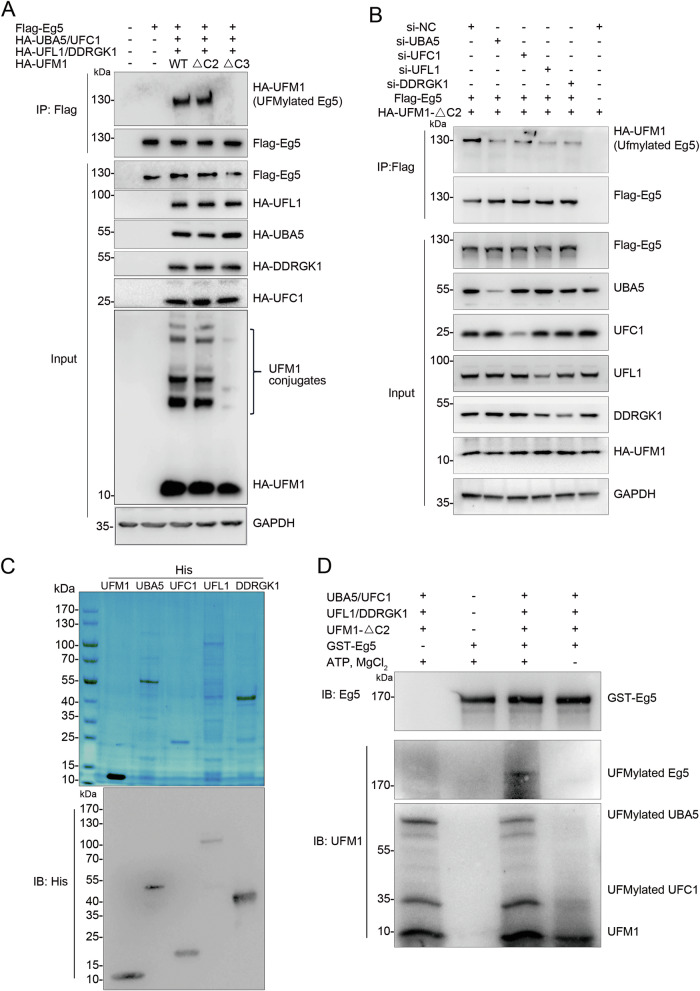
Eg5 is a target substrate for UFMylation. **A** Eg5 is UFMylated in vivo. UFMylation system components (HA-UBA5, HA-UFC1, HA-UFL1, HA-DDRGK1, and HA-UFM1) and Flag-Eg5 were expressed in HEK293T cells. Cell lysates were subjected to immunoprecipitation with anti-Flag beads followed by western blot analysis with the indicated antibodies. **B** UBA5, UFC1, UFL1, and DDRGK1 are required for Eg5 UFMylation. Flag-Eg5 and HA-UFM1-ΔC2 were expressed in UFSP2 knockout HEK293T cells with si-NC, si-UBA5, si-UFC1, si-UFL1, or si-DDRGK1, respectively. Cell lysates were subjected to the UFMylation assay. **C** Bacterially produced UFMylation components (His-UBA5, His-UFC1, His-UFL1, and His-UFM1-ΔC2) were subjected to Coomassie brilliant blue or western blot analysis with anti-His antibody. **D** Eg5 is UFMylated in vitro. Purified UFMylation components and GST-Eg5 were incubated in the UFMylation buffer. The reaction was terminated by adding an SDS sample buffer, and the samples were subjected to a western blot with the indicated antibodies.

To confirm whether Eg5 can undergo UFMylation, we co-expressed Flag-Eg5 with the UFMylation components UBA5, UFC1, UFL1, UFM1, and DDRGK1 in HEK293T cells. In vivo UFMylation assays demonstrated that Eg5 can be UFMylated by both the wild-type UFM1 (UFM1-WT) and UFM1-ΔC2, but not by the UFM1-ΔC3 (Fig. 2A). Additionally, we observed that individually knocking down UBA5, UFC1, UFL1, or DDRGK1 resulted in a decrease in the UFMylation levels of Eg5 in *UFSP2* knockout HEK293T cells (Fig. 2B), indicating their crucial involvement in the Eg5 UFMylation process. Furthermore, the UFMylation assays validated Eg5 as a target substrate for UFMylation in vitro (Fig. 2C, D). These findings unequivocally establish Eg5 as a novel target substrate for UFMylation.

### Identification of the essential UFMylation site in Eg5

**Fig. 3:**
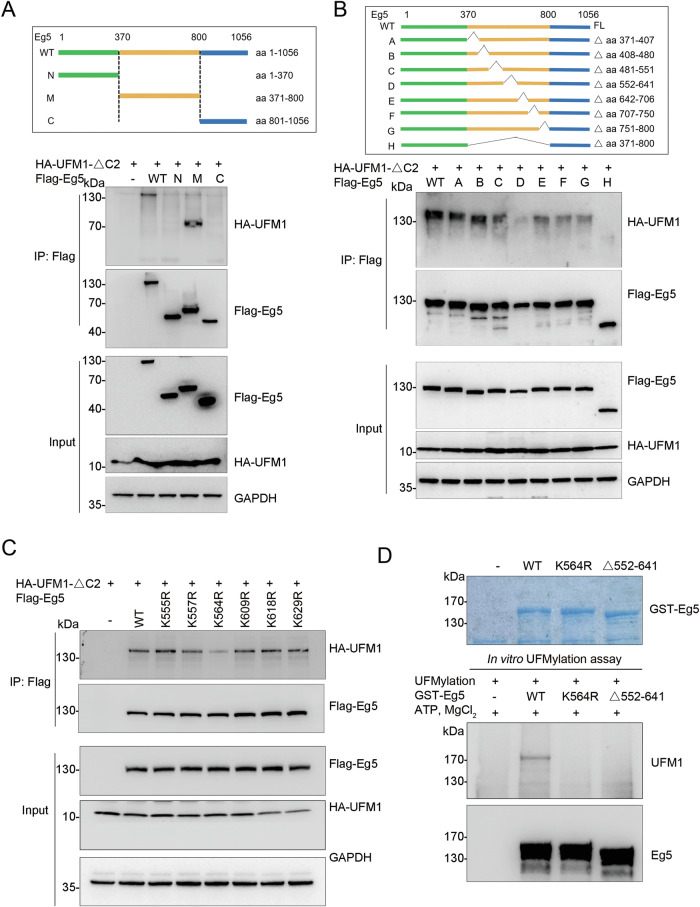
K564 is the essential UFMylation site in Eg5. **A**, **B** Identification of UFMylation region. A series of deletion constructs of Flag-Eg5 were generated as indicated, and expressed in UFSP2 knockout HEK293T cells with HA-UFM1-ΔC2. Cell lysates were subjected to the UFMylation assay. **C** Identification of the UFMylation site (s). The six Lys residues in the amino acid sequence of 552–641 were replaced by Arg, respectively, and the UFMylation assay was performed in UFSP2 knockout HEK293T cells. **D** In vitro UFMylation assay of Eg5 and its mutants, as described in Fig. 2D.

To identify the essential sites for Eg5 UFMylation, we employed three deletion constructs (ΔN, ΔM, and ΔC) and transfected them into *UFSP2* knockout HEK293T cells, along with the HA-UFM1-ΔC2 plasmid. Among these constructs, UFMylation was specifically observed in the ΔM construct of Eg5 (Fig. 3A). To further investigate the UFMylation sites, we generated additional deletion constructs targeting specific segments within the ΔM region. Notably, the deletion constructs lacking amino acids 552–641 (ΔD) showed a significant reduction in UFMylation levels (Fig. 3B). As there are six Lys residues in this region, we individually substituted each Lys residues with Arg in wild-type Eg5. Among these substitutions, the replacement of Lys564 by Arg (referred to as K564R) resulted in a diminished Eg5 UFMylation (Fig. 3C). In addition, the K564R mutation significantly reduced Eg5 UFMylation in vitro (Fig. 3D). Collectively, these data demonstrated that K564 is an essential UFMylation site in Eg5.

### UFMylation deficiency leads to decreased monoubiquitination of Eg5

**Fig. 4:**
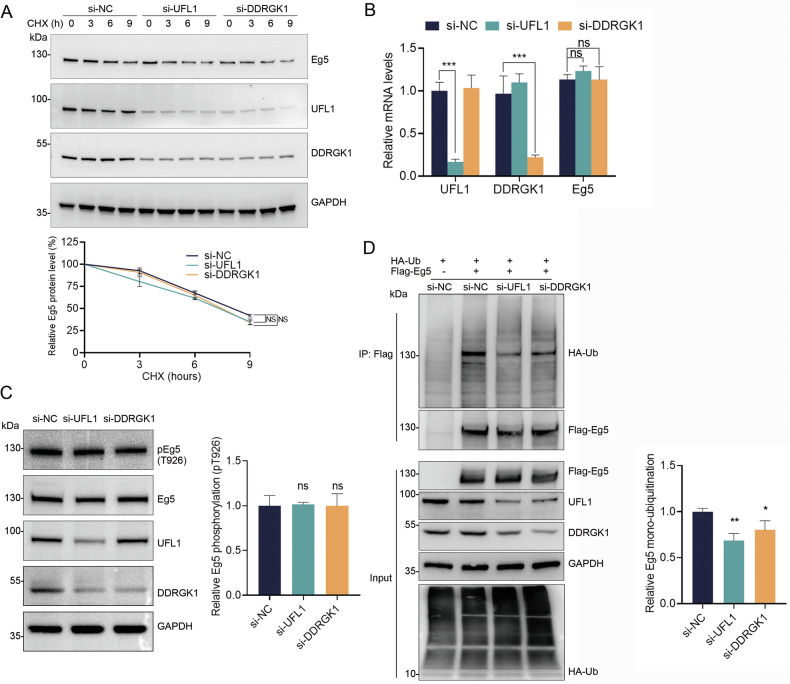
UFMylation is required for Eg5 monoubiquitination. **A** Eg5 stability was examined by western blot in HeLa cells with UFL1 or DDRGK1 knockdown. The cells were treated with 100 µg ml^−1^ cycloheximide (CHX) for the indicated times, and the Eg5 protein levels were quantified. **B** Total mRNA was extracted from HeLa cells transfected with the indicated siRNAs, and quantitative real-time RT–PCR assays were performed. **C** The phosphorylation of Eg5 at Thr926 in HeLa cells transfected with the indicated siRNAs was determined by western blot analysis. **D** HA-Ub and Flag-Eg5 were expressed in HeLa cells with si-NC, si-UFL1, or si-DDRGK1, respectively. Cell lysates were subjected to immunoprecipitation with anti-Flag beads followed by western blot analysis with the indicated antibodies. The mean ± SD from three independent experiments was shown. The *P* values were determined by one-way ANOVA. ns, not significant, *P < 0.05; **P < 0.01; ***P < 0.001.

Previous studies have shown that UFMylation is related to substrate stability [25–27]. To determine whether UFMylation influences the stability of Eg5, we suppressed the expression of UFL1 or DDRGK1 in HeLa cells using siRNAs (Fig. S2). Following treatment with cycloheximide, we observed that the stability of the Eg5 protein was not affected by the knockdown of either UFL1 or DDRGK1 (Fig. 4A). Additionally, real-time RT-PCR analysis showed that Eg5 mRNA levels remained consistent upon the depletion of UFL1 or DDRGK1 (Fig. 4B). We also examined the phosphorylation status at Thr926 on Eg5, a crucial site for post-translational modification influencing the localization of Eg5 and spindle assembly. Despite the knockdown of UFL1 or DDRGK1, the phosphorylation site (pT926) remained unaltered (Fig. 4C). Interestingly, the monoubiquitination level of Eg5 significantly decreased following UFL1 or DDRGK1 reduction (Fig. 4D), suggesting these post-translational modifications of Eg5 might play a complex role in mitosis regulation. However, we did not extensively explore how UFMylation of Eg5 affects its monoubiquitination.

### UFM1 and Eg5 exhibit co-localization at the centrosome and spindle

**Fig. 5:**
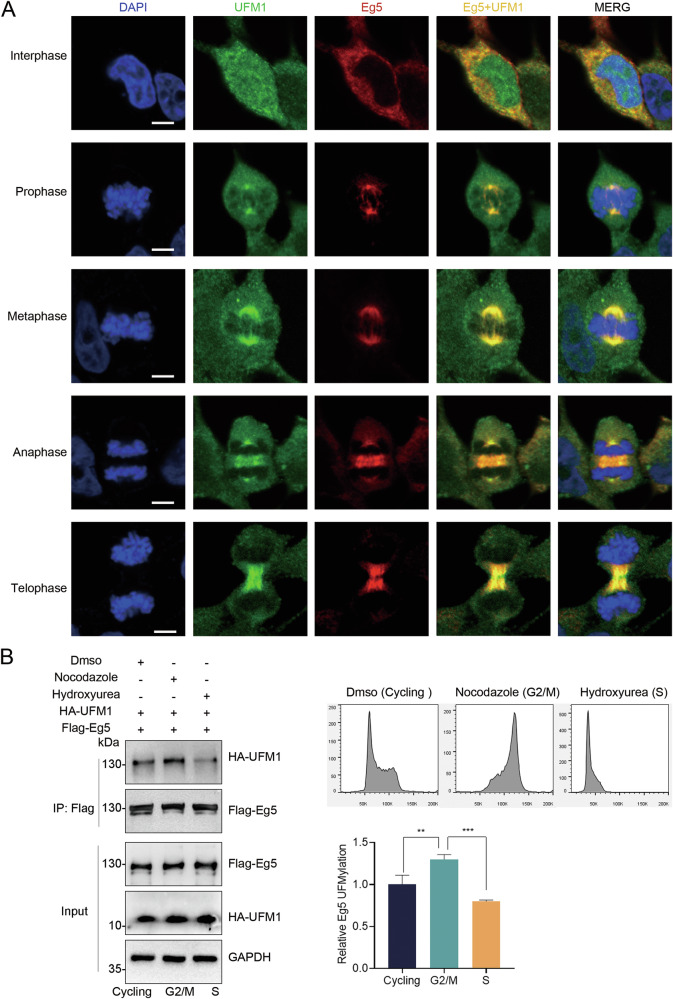
Co-localization between UFM1 and Eg5 at the centrosome and spindle. **A** The localization of UFM1 and Eg5 was detected by immunofluorescence staining using anti-UFM1 and anti-Eg5 antibodies in HeLa cells at interphase, prophase, metaphase, anaphase, and telophase. The cell nuclei were stained with DAPI. Scale bar, 5 μm. **B** HeLa cells transfected with HA-UFM1 and Flag-Eg5 were treated with DMSO, Hydroxyurea, and Nocodazole, respectively. The cell lysates were then subjected to a UFMylation assay. The mean ± SD from three independent experiments was shown. The *P* values were determined by one-way ANOVA. **P < 0.01; ***P < 0.001.

To gain functional insight into the association between UFMylation and Eg5, we investigated the cellular localization of UFM1 and Eg5. Immunofluorescence staining revealed that during interphase, UFM1 was present in both the nucleus and the cytoplasm, while Eg5 was predominantly localized in the cytoplasm. As cells entered prophase, UFM1 and Eg5 started to accumulate at the centrosomes, displaying an overlapping pattern. During metaphase, UFM1 and Eg5 were distributed throughout the spindle apparatus. Subsequently, UFM1 and Eg5 were observed at the spindle poles and midbody in anaphase and telophase (Fig. 5A). To investigate whether Eg5 UFMylation is restricted during the cell cycle, we assessed the levels of Eg5 UFMylation after synchronizing HeLa cells in the S phase or G2/M phase using Hydroxyurea and Nocodazole. It showed that Eg5 UFMylation primarily occurs during mitosis (Fig. 5B). The dynamic co-localization and the temporal specificity of the modification suggest their potential roles in mitotic processes and spindle organization.

### UFMylation maintains Eg5 interaction with the mitotic spindle

**Fig. 6:**
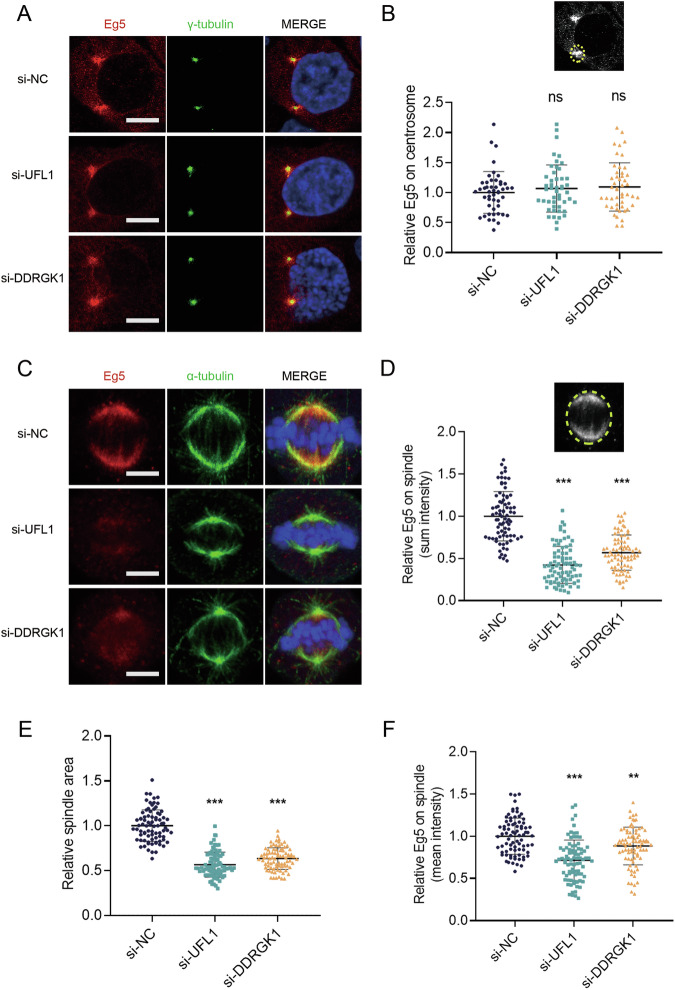
Defective Eg5 UFMylation impairs the distribution of Eg5 on the mitotic spindle. **A** The localization of Eg5 at the centrosome was detected by immunofluorescence staining using anti-Eg5 and anti-γ-tubulin in HeLa cells. Scale bar, 5 μm. The mean ± SD from at least 45 mitotic cells was shown. The *P* values were determined by one-way ANOVA. ns, not significant. **B** Sum intensity of Eg5 in the prophase centrosome region. **C** The localization of Eg5 at the spindle was detected by immunofluorescence staining using anti-Eg5 and anti-α-tubulin in HeLa cells. **D**–**F** Sum intensity of Eg5, spindle area, and mean intensity of Eg5 in the metaphase spindle region. Scale bar, 5 μm. The mean ± SD from at least 80 mitotic cells was shown. The *P* values were determined by one-way ANOVA. *P < 0.05; **P < 0.01; ***P < 0.001.

The spatial and temporal dynamics of Eg5 are crucial for its motor function in driving centrosome separation, spindle assembly, and chromosome segregation during mitosis [24]. Given the co-localization of UFM1 and Eg5 at the centrosome and spindle, we were intrigued to explore whether UFMylation affects the spatial relationship between Eg5 and the mitotic centrosome or spindle. Indeed, the knockdown of UFL1 or DDRGK1 had no influence on the localization of Eg5 at the centrosome during prophase (Fig. 6A, B). However, the knockdown of UFL1 or DDRGK1 led to a smaller spindle area and a reduction in both sun and mean Eg5 intensity at the spindle during metaphase (Fig. 6C–F). These results suggested that Eg5 UFMylation is crucial for maintaining its interaction with the mitotic spindle.

### UFMylation promotes spindle organization, cell cycle progression, and cell proliferation

**Fig. 7:**
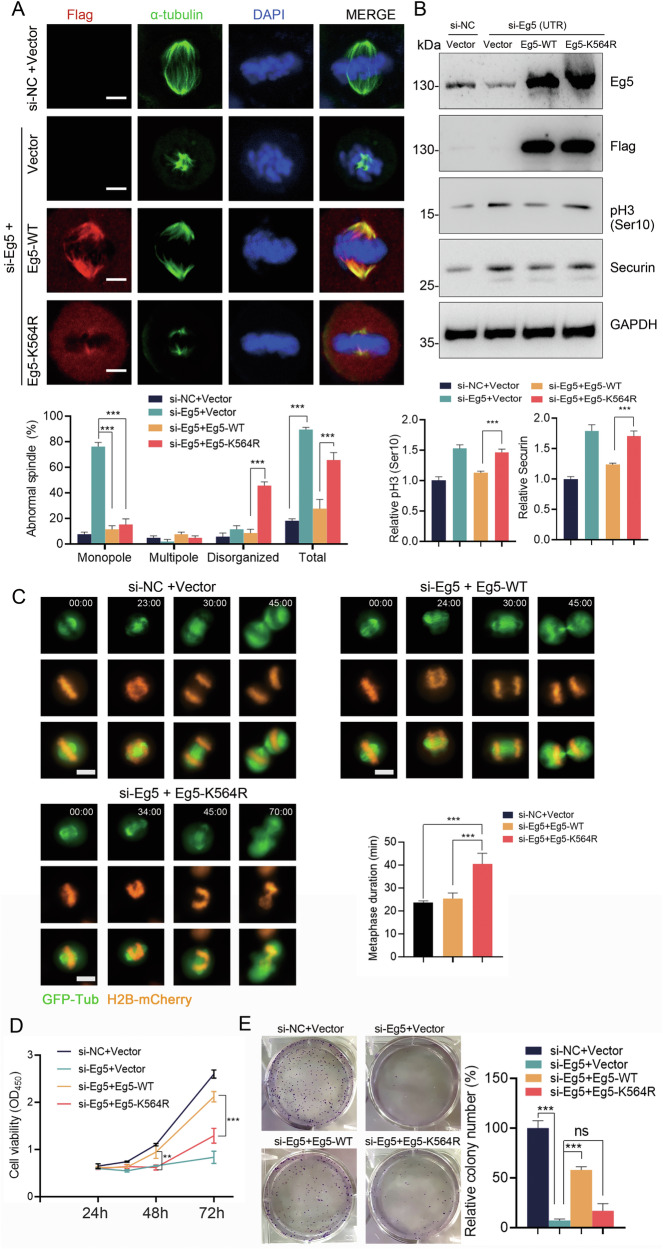
Eg5 UFMylation is required for the spindle assembly and cell proliferation. HeLa cells were transfected with indicated siRNAs or plasmids. **A** The phenotypes of the mitotic spindle (monopole, multipole, or disorganized) were determined by immunofluorescence staining using anti-α-tubulin and DAPI in HeLa cells. Transfected cells were identified by Flag positivity. The percent of mitotic phenotypes was calculated. Scale bar, 5 μm. The mean ± SD from three independent experiments, with 100 cells per experimental group, was shown. The *P* values were determined by two-way ANOVA. ***P < 0.001. **B** Cell lysates were subjected to western blot analysis with the indicated antibodies. The mean ± SD from three independent experiments was shown. The *P* values were determined by one-way ANOVA. ***P < 0.001. **C** Time-lapse imaging of HeLa cells expressing H2B-mCherry and GFP-Tub was conducted following co-transfection with the indicated siRNAs or plasmids. Time was shown as min:s. Scale bar, 10 μm. The duration of metaphase was shown: si-NC+Vector, *n* = 15; si-Eg5 + Eg5-WT, *n* = 15; si-NC + Eg5-K564R (*n* = 25, 15 arrested in metaphase, 10 quantified). The *P* values were determined by one-way ANOVA. ***P < 0.001. **D** HeLa cells were cultured for 24 h, 36 h, 48 h, and 72 h, then subjected to CCK-8 assays. The mean ± SD from three independent experiments was shown. The *P* values were determined by one-way ANOVA. **P < 0.01; ***P < 0.001. **E** Colony formation assay. HeLa cells were cultured and stained with crystal violet. The number of colonies in each condition was counted. The mean ± SD from three independent experiments was shown. The *P* values were determined by one-way ANOVA. ***P < 0.001.

Given the crucial role of Eg5 in mitotic spindle assembly, we proceeded to explore the effects of Eg5 UFMylation on mitotic spindle morphology. We employed siRNAs to suppress Eg5 expression in HeLa cells and observed a significant increase in mitotic arrest, characterized by condensed chromosomes arranged around a monopolar spindle (Fig. S3), a phenomenon also observed in other studies [28, 29]. Next, we co-transfected Flag-Eg5-WT or Flag-Eg5-K564R plasmids to rescue the effects of Eg5 siRNA-3, which targets the 3’-UTR sequence without affecting the expression of exogenous Eg5 (Fig. 7B). Remarkably, spindle bipolarity was restored in HeLa cells co-transfected with Eg5 siRNA and Eg5-WT. In contrast, cells co-transfected with Eg5 siRNA and Eg5-K564R displayed shortened spindles (Fig. 7A). To delve deeper into the mitotic phenotypes caused by the loss of Eg5 UFMylation, we employed time-lapse imaging to track chromosomes and spindles with H2B-mCherry and GFP-Tub. The results showed that cells co-transfected with Eg5 siRNA and rescued with Eg5-WT were able to proceed through mitosis effectively. Conversely, among the 25 cells rescued with the Eg5-K564R mutant, 15 cells were arrested in metaphase for the entire 90-min imaging period. For the remaining 10 cells, it took 34–45 min to complete metaphase (Fig. 7C). An increase in the mitotic marker pH3 (Ser10) and the spindle checkpoint marker Securin was noted (Fig. 7B), indicating the activation of the spindle checkpoint, which likely contributes to the failure of the mitotic process. Additionally, CCK-8 and colony formation assays showed that the knockdown of Eg5 significantly inhibited cell proliferation in HeLa cells (Fig. 7D, E). This inhibitory effect was rescued by the co-expression of Eg5-WT, whereas the co-expression of Eg5-K564R failed to produce the same rescue effect. Collectively, these data demonstrated that Eg5 UFMylation plays a critical role in promoting spindle organization, mitotic progression, and cell proliferation.

## Discussion

In our previous research, we demonstrated that a deficiency in UFMylation significantly disrupts the process of mitosis [22]. The present study introduces Eg5 as a novel substrate, emerging as a highly plausible candidate based on our and other proteomics data [25, 27, 30, 31]. This finding established a direct link between UFMylation and the regulation of mitosis.

We found that Eg5 UFMylation occurs predominantly during mitosis, where Eg5 plays an essential role in spindle formation by generating forces that establish and maintain spindle bipolarity and contribute to spindle elongation. Throughout mitosis, Eg5 undergoes post-translational modifications, including phosphorylation, ubiquitination, and acetylation, at specific sites, each serving specialized roles at different mitotic stages [23, 24]. During prophase, Eg5 is primarily located near the centrosome, facilitating centrosome separation and spindle formation by promoting the sliding of surrounding microtubules. Its interaction with TPX2 contributes to its localization to the centrosome [32]. Our results showed the loss of UFMylation did not noticeably affect Eg5’s localization at the centrosome in prophase or prometaphase, nor did it result in a monopolar spindle phenotype. UFMylation did not alter the phosphorylation site pT926, which is crucial for Eg5’s localization and spindle assembly during prophase. During metaphase, Eg5 predominantly localizes to the spindle, where it crosslinks microtubule proteins, stabilizes the bipolar structure, and participates in the dynamic adjustment of the spindle. Our findings revealed that UFM1 and Eg5 co-localized on the spindle, and the loss of UFMylation led to a significant reduction in Eg5’s spindle localization, resulting in spindle shortening. Time-lapse imaging showed that mitosis was arrested at metaphase. Abnormal spindle morphology activated the SAC, offering cells a chance to correct spindle anomalies. An increase in Securin levels, reflective of prolonged SAC activation, was observed in the UFMylation-deficient K564R mutant, suggesting a mitotic delay and, consequently, a failure of cell proliferation. These results demonstrated that UFMylation of Eg5 mainly functions during the metaphase stage, contributing to the assembly and maintenance of the spindle.

Although UFMylation was discovered about two decades ago, the majority of its substrates have only been identified in recent years [10, 33]. Research into these substrates has revealed a broad spectrum of functions associated with this modification. For example, UFMylation of RPL26, RPN1, CYB5R3, and HRD1 maintains the ER proteins during ER stress [31, 34–37]. UFMylation regulates tumorigenesis through ASC1, SLC7A11, and PD-L1 [38–40]. P4HB UFMylation impacts mitochondrial oxidative stress [27]. Atg9 UFMylation protects the nerves [41]. UFMylation of MRE11, histone H4, and p53 triggers DNA damage response following double-strand breaks [25, 42, 43]. DNA damage can occur at different stages of mitosis and may contribute to mitotic abnormalities. The occurrence of DNA damage during the G1, S, or G2 phases activates the DNA damage checkpoint, resulting in the arrest of the cell cycle in interphase. If damage arises during prophase and metaphase, cells pass through anaphase and telophase without arrest, resulting in activation of the DNA damage checkpoint in the subsequent G1 phase [44, 45]. Nonetheless, our observations indicate that the knockdown of either UFL1 or DDRGK1 predominantly arrests the cell cycle at metaphase, suggesting that DNA damage may not be the direct cause of mitotic defects upon UFMylation deficiency. Instead, our findings reveal that the loss of Eg5 UFMylation led to the formation of shortened or misaligned metaphase spindles, demonstrating its importance as a primary substrate of UFMylation during the mitotic process.

During mitosis, the localization and function of Eg5 are intricately regulated by post-translational modifications. Phosphorylation, acetylation, and monoubiquitination of Eg5 are known to play roles in spindle assembly and centrosomal dynamics [29, 46–50]. Our data reveal that UFMylation at the K564 site is crucial for ensuring the correct localization of Eg5 on the spindle, a step essential for the assembly and maintenance of the spindle. Additionally, impaired UFMylation was found to decrease the monoubiquitination levels of Eg5, suggesting a complex interplay among various post-translational modifications. A similar phenomenon has been observed in p53, another substrate for UFMylation. UFMylation at the K351, K357, K370, and K373 sites of p53 can inhibit its polyubiquitination, thereby enhancing its protein stability. Furthermore, these sites may also undergo acetylation or ubiquitination modifications, but the regulatory mechanisms between these modifications remain elusive [25]. In conclusion, the precise post-translational modifications of Eg5 ensure its optimal function throughout the complexities of mitosis.

Our study elucidates that UFMylation of Eg5 promotes spindle assembly during mitosis, providing insights into the molecular pathogenesis of microcephaly. It’s noteworthy that individuals with mutations in *Eg5* typically exhibit severe microcephaly and developmental delays [51–53], which closely resemble the phenotypes observed in patients with mutations in *UBA5*, *UFM1*, or *UFC1*. According to the gnomAD database, the allele frequency of c.1111 G > A (p.A371T) in *UBA5* is reported as 0.0027, with a prevalence of over 60% in patients carrying this variant. Future research could explore precision therapies, similar to those developed for various CFTR mutations [54], which may help maintain UFMylation modification on critical mitotic substrates like Eg5 or even other substrates, thereby facilitating the development of effective treatment strategies.

Furthermore, a recent study has unveiled the cryo-electron microscopy structure of the UFMylation E3 ligase complexed with its substrate RPL26, marking an advancement in our understanding of the complex molecular dynamics within the UFMylation pathway [55]. Investigating these interactions between substrates and the UFMylation system reveals promising strategies to maintain the modification activity of Eg5, thereby offering potential therapeutic avenues for related diseases.

### Supplementary information

Dataset 1

Appendix tables and supplementary figures

Original WB Data

## Data Availability

The data generated in this study are available upon request from the corresponding author.

## References

[1] Komatsu M, Chiba T, Tatsumi K, Iemura S, Tanida I, Okazaki N, et al. A novel protein-conjugating system for Ufm1, a ubiquitin-fold modifier. Embo J. 2004;23:1977–86. 15071506 10.1038/sj.emboj.7600205 PMC404325

[2] Gerakis Y, Quintero M, Li H, Hetz C. The UFMylation system in proteostasis and beyond. Trends Cell Biol. 2019;29:974–86. 31703843 10.1016/j.tcb.2019.09.005 PMC6917045

[3] Banerjee S, Kumar M, Wiener R. Decrypting UFMylation: how proteins are modified with UFM1. Biomolecules. 2020;10:1442. 33066455 10.3390/biom10101442 PMC7602216

[4] Kang SH, Kim GR, Seong M, Baek SH, Seol JH, Bang OS, et al. Two novel ubiquitin-fold modifier 1 (Ufm1)-specific proteases, UfSP1 and UfSP2. J Biol Chem. 2007;282:5256–62. 17182609 10.1074/jbc.M610590200

[5] Bacik JP, Walker JR, Ali M, Schimmer AD, Dhe-Paganon S. Crystal structure of the human ubiquitin-activating enzyme 5 (UBA5) bound to ATP: mechanistic insights into a minimalistic E1 enzyme. J Biol Chem. 2010;285:20273–80. 20368332 10.1074/jbc.M110.102921 PMC2888440

[6] Liu G, Forouhar F, Eletsky A, Atreya HS, Aramini JM, Xiao R, et al. NMR and X-RAY structures of human E2-like ubiquitin-fold modifier conjugating enzyme 1 (UFC1) reveal structural and functional conservation in the metazoan UFM1-UBA5-UFC1 ubiquination pathway. J Struct Funct Genom. 2009;10:127–36. 10.1007/s10969-008-9054-7 PMC2850604 19101823

[7] Tatsumi K, Sou YS, Tada N, Nakamura E, Iemura S, Natsume T, et al. A novel type of E3 ligase for the Ufm1 conjugation system. J Biol Chem. 2010;285:5417–27. 20018847 10.1074/jbc.M109.036814 PMC2820770

[8] Wu J, Lei G, Mei M, Tang Y, Li H. A novel C53/LZAP-interacting protein regulates stability of C53/LZAP and DDRGK domain-containing Protein 1 (DDRGK1) and modulates NF-kappaB signaling. J Biol Chem. 2010;285:15126–36. 20228063 10.1074/jbc.M110.110619 PMC2865345

[9] Witting KF, Mulder M. Highly specialized ubiquitin-like modifications: shedding light into the UFM1 enigma. Biomolecules. 2021;11:255. 33578803 10.3390/biom11020255 PMC7916544

[10] Zhou X, Mahdizadeh SJ, Le Gallo M, Eriksson LA, Chevet E, Lafont E. UFMylation: a ubiquitin-like modification. Trends Biochem Sci. 2023. 10.1016/j.tibs.2023.10.004 10.1016/j.tibs.2023.10.004 37945409

[11] Chung CH, Yoo HM. Emerging role of protein modification by UFM1 in cancer. Biochem Biophys Res Commun. 2022;633:61–63. 36344165 10.1016/j.bbrc.2022.08.093

[12] Cheng Y, Niu Z, Cai Y, Zhang W. Emerging role of UFMylation in secretory cells involved in the endocrine system by maintaining ER proteostasis. Front Endocrinol. 2022;13:1085408. 10.3389/fendo.2022.1085408 PMC9894094 36743909

[13] Duan R, Shi Y, Yu L, Zhang G, Li J, Lin Y, et al. UBA5 mutations cause a new form of autosomal recessive cerebellar ataxia. Plos One. 2016;11:e149039. 10.1371/journal.pone.0149039 PMC4752235 26872069

[14] Colin E, Daniel J, Ziegler A, Wakim J, Scrivo A, Haack TB, et al. Biallelic variants in UBA5 reveal that disruption of the UFM1 cascade can result in early-onset encephalopathy. Am J Hum Genet. 2016;99:695–703. 27545681 10.1016/j.ajhg.2016.06.030 PMC5011045

[15] Muona M, Ishimura R, Laari A, Ichimura Y, Linnankivi T, Keski-Filppula R, et al. Biallelic variants in UBA5 link dysfunctional UFM1 ubiquitin-like modifier pathway to severe infantile-onset encephalopathy. Am J Hum Genet. 2016;99:683–94. 27545674 10.1016/j.ajhg.2016.06.020 PMC5010641

[16] Arnadottir GA, Jensson BO, Marelsson SE, Sulem G, Oddsson A, Kristjansson RP, et al. Compound heterozygous mutations in UBA5 causing early-onset epileptic encephalopathy in two sisters. BMC Med Genet. 2017;18:103. 28965491 10.1186/s12881-017-0466-8 PMC5623963

[17] Tumiene B, Maver A, Writzl K, Hodzic A, Cuturilo G, Kuzmanic-Samija R, et al. Diagnostic exome sequencing of syndromic epilepsy patients in clinical practice. Clin Genet. 2018;93:1057–62. 29286531 10.1111/cge.13203

[18] Daida A, Hamano SI, Ikemoto S, Matsuura R, Nakashima M, Matsumoto N, et al. Biallelic loss-of-function UBA5 mutations in a patient with intractable West syndrome and profound failure to thrive. Epileptic Disord. 2018;20:313–18. 30078785 10.1684/epd.2018.0981

[19] Low KJ, Baptista J, Babiker M, Caswell R, King C, Ellard S, et al. Hemizygous UBA5 missense mutation unmasks recessive disorder in a patient with infantile-onset encephalopathy, acquired microcephaly, small cerebellum, movement disorder and severe neurodevelopmental delay. Eur J Med Genet. 2019;62:97–102. 29902590 10.1016/j.ejmg.2018.06.009

[20] Hamilton E, Bertini E, Kalaydjieva L, Morar B, Dojcakova D, Liu J, et al. UFM1 founder mutation in the Roma population causes recessive variant of H-ABC. Neurology. 2017;89:1821–28. 28931644 10.1212/WNL.0000000000004578 PMC5664304

[21] Nahorski MS, Maddirevula S, Ishimura R, Alsahli S, Brady AF, Begemann A, et al. Biallelic UFM1 and UFC1 mutations expand the essential role of ufmylation in brain development. Brain. 2018;141:1934–45. 29868776 10.1093/brain/awy135 PMC6022668

[22] Yu L, Li G, Deng J, Jiang X, Xue J, Zhu Y, et al. The UFM1 cascade times mitosis entry associated with microcephaly. Faseb J. 2020;34:1319–30. 31914610 10.1096/fj.201901751R

[23] Liu M, Ran J, Zhou J. Non-canonical functions of the mitotic kinesin Eg5. Thorac Cancer. 2018;9:904–10. 29927078 10.1111/1759-7714.12792 PMC6068462

[24] Mann BJ, Wadsworth P.Kinesin-5 regulation and function in mitosis.Trends Cell Biol. 2019;29:66–79. 30220581 10.1016/j.tcb.2018.08.004

[25] Liu J, Guan D, Dong M, Yang J, Wei H, Liang Q, et al. UFMylation maintains tumour suppressor p53 stability by antagonizing its ubiquitination. Nat Cell Biol. 2020;22:1056–63. 32807901 10.1038/s41556-020-0559-z

[26] Yoo HM, Park JH, Kim JY, Chung CH. Modification of ERalpha by UFM1 increases its stability and transactivity for breast cancer development. Mol Cells. 2022;45:425–34. 35680375 10.14348/molcells.2022.0029 PMC9200662

[27] Zhu J, Ma X, Jing Y, Zhang G, Zhang D, Mao Z, et al. P4HB UFMylation regulates mitochondrial function and oxidative stress. Free Radic Biol Med. 2022;188:277–86. 35753586 10.1016/j.freeradbiomed.2022.06.237

[28] Blangy A, Lane HA, D’Herin P, Harper M, Kress M, Nigg EA. Phosphorylation by p34cdc2 regulates spindle association of human Eg5, a kinesin-related motor essential for bipolar spindle formation in vivo. Cell. 1995;83:1159–69. 8548803 10.1016/0092-8674(95)90142-6

[29] Zheng J, Tan Y, Liu X, Zhang C, Su K, Jiang Y, et al. NAT10 regulates mitotic cell fate by acetylating Eg5 to control bipolar spindle assembly and chromosome segregation. Cell Death Differ. 2022;29:846–60. 35210604 10.1038/s41418-021-00899-5 PMC8989979

[30] Pirone L, Xolalpa W, Sigurethsson JO, Ramirez J, Perez C, Gonzalez M, et al. A comprehensive platform for the analysis of ubiquitin-like protein modifications using in vivo biotinylation. Sci Rep. 2017;7:40756. 28098257 10.1038/srep40756 PMC5241687

[31] Ishimura R, El-Gowily AH, Noshiro D, Komatsu-Hirota S, Ono Y, Shindo M, et al. The UFM1 system regulates ER-phagy through the ufmylation of CYB5R3. Nat Commun. 2022;13:7857. 36543799 10.1038/s41467-022-35501-0 PMC9772183

[32] Ma N, Titus J, Gable A, Ross JL, Wadsworth P. TPX2 regulates the localization and activity of Eg5 in the mammalian mitotic spindle. J Cell Biol. 2011;195:87–98. 21969468 10.1083/jcb.201106149 PMC3187703

[33] Wang X, Xu X, Wang Z. The post-translational role of UFMylation in physiology and disease. Cells. 2023;12:2543. 37947621 10.3390/cells12212543 PMC10648299

[34] Walczak CP, Leto DE, Zhang L, Riepe C, Muller RY, DaRosa PA, et al. Ribosomal protein RPL26 is the principal target of UFMylation. Proc Natl Acad Sci USA. 2019;116:1299–308. 30626644 10.1073/pnas.1816202116 PMC6347690

[35] Wang L, Xu Y, Rogers H, Saidi L, Noguchi CT, Li H, et al. UFMylation of RPL26 links translocation-associated quality control to endoplasmic reticulum protein homeostasis. Cell Res. 2020;30:5–20. 31595041 10.1038/s41422-019-0236-6 PMC6951344

[36] Liang JR, Lingeman E, Luong T, Ahmed S, Muhar M, Nguyen T, et al. A genome-wide ER-phagy screen highlights key roles of mitochondrial metabolism and ER-resident UFMylation. Cell. 2020;180:1160–77. 32160526 10.1016/j.cell.2020.02.017 PMC7197389

[37] Luo H, Jiao QB, Shen CB, Gong WY, Yuan JH, Liu YY, et al. UFMylation of HRD1 regulates endoplasmic reticulum homeostasis. Faseb J. 2023;37:e23221. 37795761 10.1096/fj.202300004RRRR

[38] Yoo HM, Kang SH, Kim JY, Lee JE, Seong MW, Lee SW, et al. Modification of ASC1 by UFM1 is crucial for ERalpha transactivation and breast cancer development. Mol Cell. 2014;56:261–74. 25219498 10.1016/j.molcel.2014.08.007

[39] Yang J, Zhou Y, Xie S, Wang J, Li Z, Chen L, et al. Metformin induces ferroptosis by inhibiting UFMylation of SLC7A11 in breast cancer. J Exp Clin Cancer Res. 2021;40:206. 34162423 10.1186/s13046-021-02012-7 PMC8223374

[40] Zhou J, Ma X, He X, Chen B, Yuan J, Jin Z, et al. Dysregulation of PD-L1 by UFMylation imparts tumor immune evasion and identified as a potential therapeutic target. Proc Natl Acad Sci USA. 2023;120:e2079235176. 10.1073/pnas.2215732120 PMC10089188 36893266

[41] Li H, Yu Z, Niu Z, Cheng Y, Wei Z, Cai Y, et al. A neuroprotective role of Ufmylation through Atg9 in the aging brain of Drosophila. Cell Mol Life Sci. 2023;80:129. 37086384 10.1007/s00018-023-04778-9 PMC11073442

[42] Qin B, Yu J, Nowsheen S, Wang M, Tu X, Liu T, et al. UFL1 promotes histone H4 ufmylation and ATM activation. Nat Commun. 2019;10:1242. 30886146 10.1038/s41467-019-09175-0 PMC6423285

[43] Wang Z, Gong Y, Peng B, Shi R, Fan D, Zhao H, et al. MRE11 UFMylation promotes ATM activation. Nucleic Acids Res. 2019;47:4124–35. 30783677 10.1093/nar/gkz110 PMC6486557

[44] Hayashi MT, Karlseder J. DNA damage associated with mitosis and cytokinesis failure. Oncogene. 2013;32:4593–601. 23318447 10.1038/onc.2012.615 PMC3681845

[45] Heijink AM, Krajewska M, van Vugt MA. The DNA damage response during mitosis. Mutat Res. 2013;750:45–55. 23880065 10.1016/j.mrfmmm.2013.07.003

[46] Rapley J, Nicolas M, Groen A, Regue L, Bertran MT, Caelles C, et al. The NIMA-family kinase Nek6 phosphorylates the kinesin Eg5 at a novel site necessary for mitotic spindle formation. J Cell Sci. 2008;121:3912–21. 19001501 10.1242/jcs.035360 PMC4066659

[47] Smith E, Hégarat N, Vesely C, Roseboom I, Larch C, Streicher H, et al. Differential control of Eg5-dependent centrosome separation by Plk1 and Cdk1. Embo J. 2011;30:2233–45. 21522128 10.1038/emboj.2011.120 PMC3117641

[48] Duan Y, Huo D, Gao J, Wu H, Ye Z, Liu Z, et al. Ubiquitin ligase RNF20/40 facilitates spindle assembly and promotes breast carcinogenesis through stabilizing motor protein Eg5. Nat Commun. 2016;7:12648. 27557628 10.1038/ncomms12648 PMC5007379

[49] Bickel KG, Mann BJ, Waitzman JS, Poor TA, Rice SE, Wadsworth P. Src family kinase phosphorylation of the motor domain of the human kinesin-5, Eg5. Cytoskeleton. 2017;74:317–30. 28646493 10.1002/cm.21380 PMC5735839

[50] Muretta JM, Reddy B, Scarabelli G, Thompson AF, Jariwala S, Major J, et al. A posttranslational modification of the mitotic kinesin Eg5 that enhances its mechanochemical coupling and alters its mitotic function. Proc Natl Acad Sci USA. 2018;115:E1779–88. 29432173 10.1073/pnas.1718290115 PMC5828613

[51] Hazan F, Ostergaard P, Ozturk T, Kantekin E, Atlihan F, Jeffery S, et al. A novel KIF11 mutation in a Turkish patient with microcephaly, lymphedema, and chorioretinal dysplasia from a consanguineous family. Am J Med Genet A. 2012;158A:1686–89. 22653704 10.1002/ajmg.a.35371

[52] Mirzaa GM, Enyedi L, Parsons G, Collins S, Medne L, Adams C, et al. Congenital microcephaly and chorioretinopathy due to de novo heterozygous KIF11 mutations: five novel mutations and review of the literature. Am J Med Genet A. 2014;164A:2879–86. 25115524 10.1002/ajmg.a.36707 PMC4205200

[53] Jones GE, Ostergaard P, Moore AT, Connell FC, Williams D, Quarrell O, et al. Microcephaly with or without chorioretinopathy, lymphoedema, or mental retardation (MCLMR): review of phenotype associated with KIF11 mutations. Eur J Hum Genet. 2014;22:881–87. 24281367 10.1038/ejhg.2013.263 PMC3938398

[54] Lopes-Pacheco M. CFTR modulators: the changing face of cystic fibrosis in the era of precision medicine. Front Pharm. 2019;10:1662. 10.3389/fphar.2019.01662 PMC7046560 32153386

[55] Makhlouf L, Peter JJ, Magnussen HM, Thakur R, Millrine D, Minshull TC, et al. The UFM1 E3 ligase recognizes and releases 60S ribosomes from ER translocons. Nature. 2024;627:437–44. 38383789 10.1038/s41586-024-07093-w PMC10937380

