# Supplementary material for: Eg5 UFMylation promotes spindle organization during mitosis
Source: Cell Death Dis. 2024 Jul 31;15(7):544. doi: 10.1038/s41419-024-06934-w (PMC11291904; doi:10.1038/s41419-024-06934-w)
Supplement: Supplementary file 2 — Appendix tables and supplementary figures [file 41419_2024_6934_MOESM2_ESM.doc]

**Appendix tables and supplementary figures**

**TABLE S1.** Mass spectrometry information of UFM1 enrichment. The immunoprecipitates of Flag-UFM1-ΔC2 or Flag-UFM1ΔC3 were resolved by SDS-PAGE and subjected to mass spectrometry. The protein accession, description, coverage, unique peptide and molecular weight are listed.

**FIGURE S1.** UFSP2 KO resulted in increased levels of protein UFMylation.


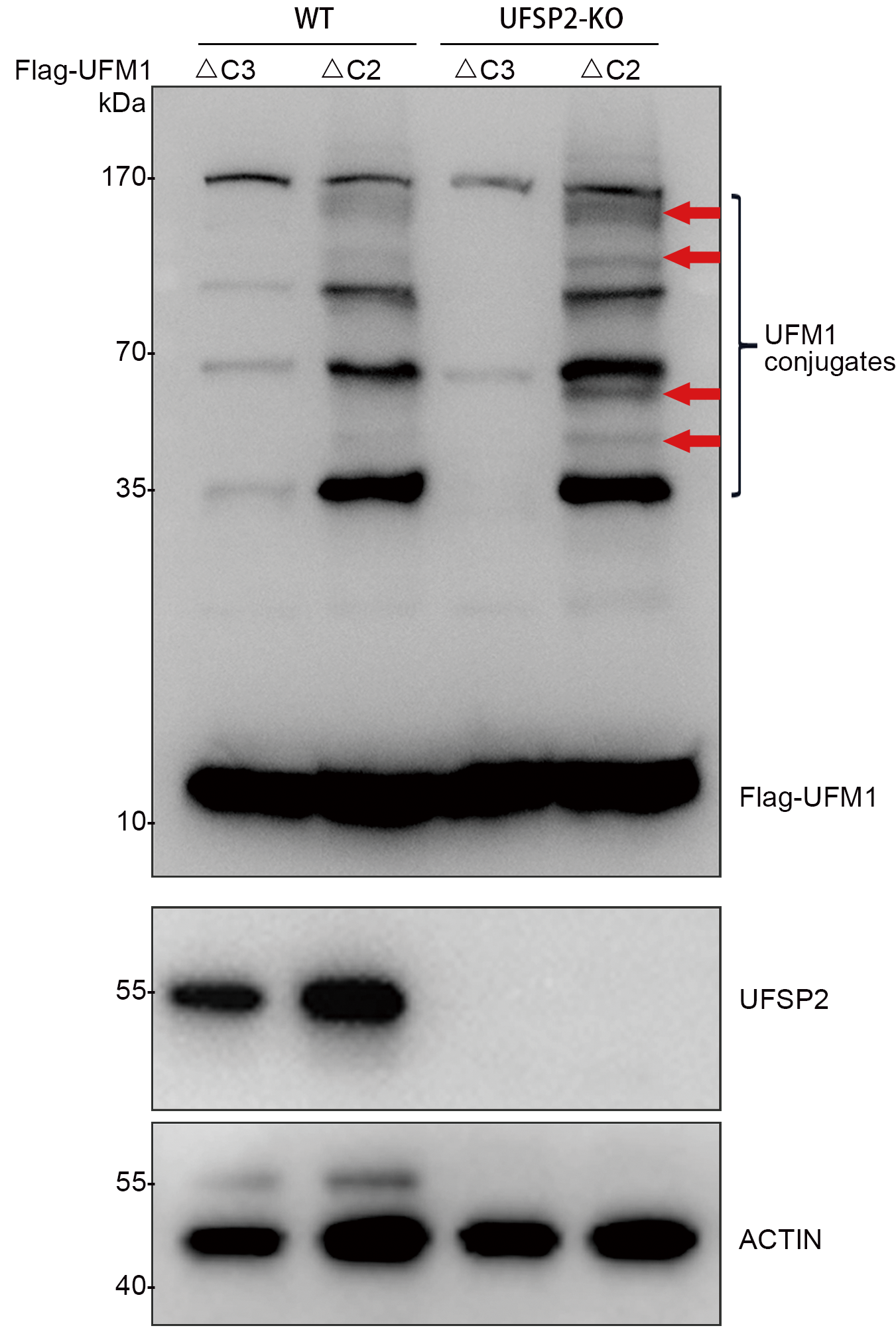

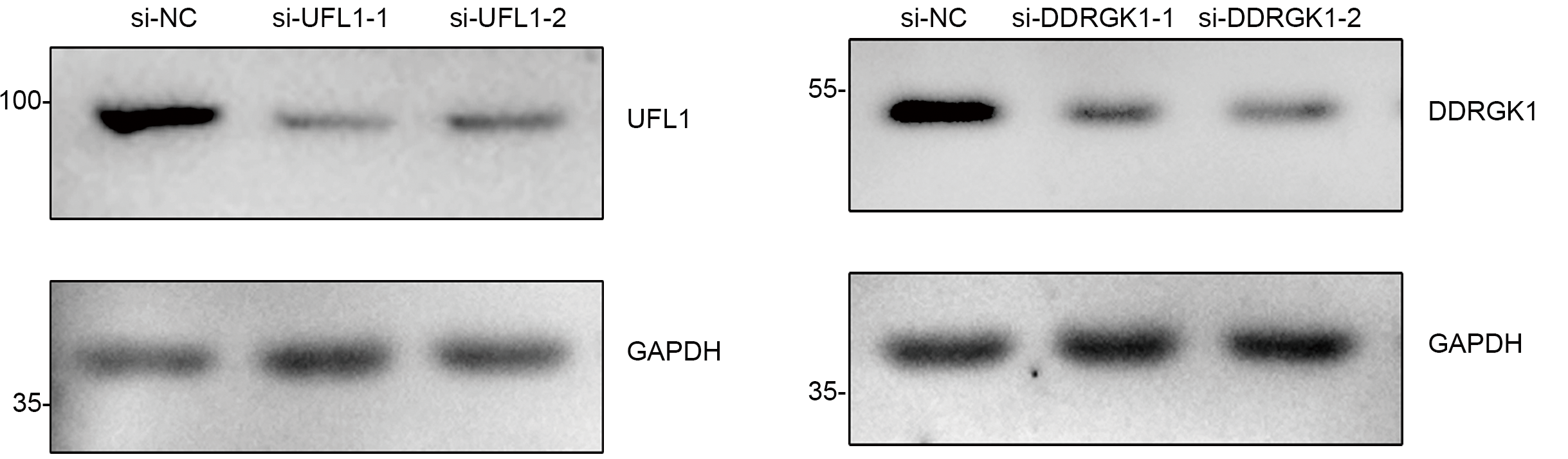
**FIGURE S2.** The expression of UFL1 or DDRGK1 in HeLa cells transfected with indicated siRNAs was determined by Western blot analysis.


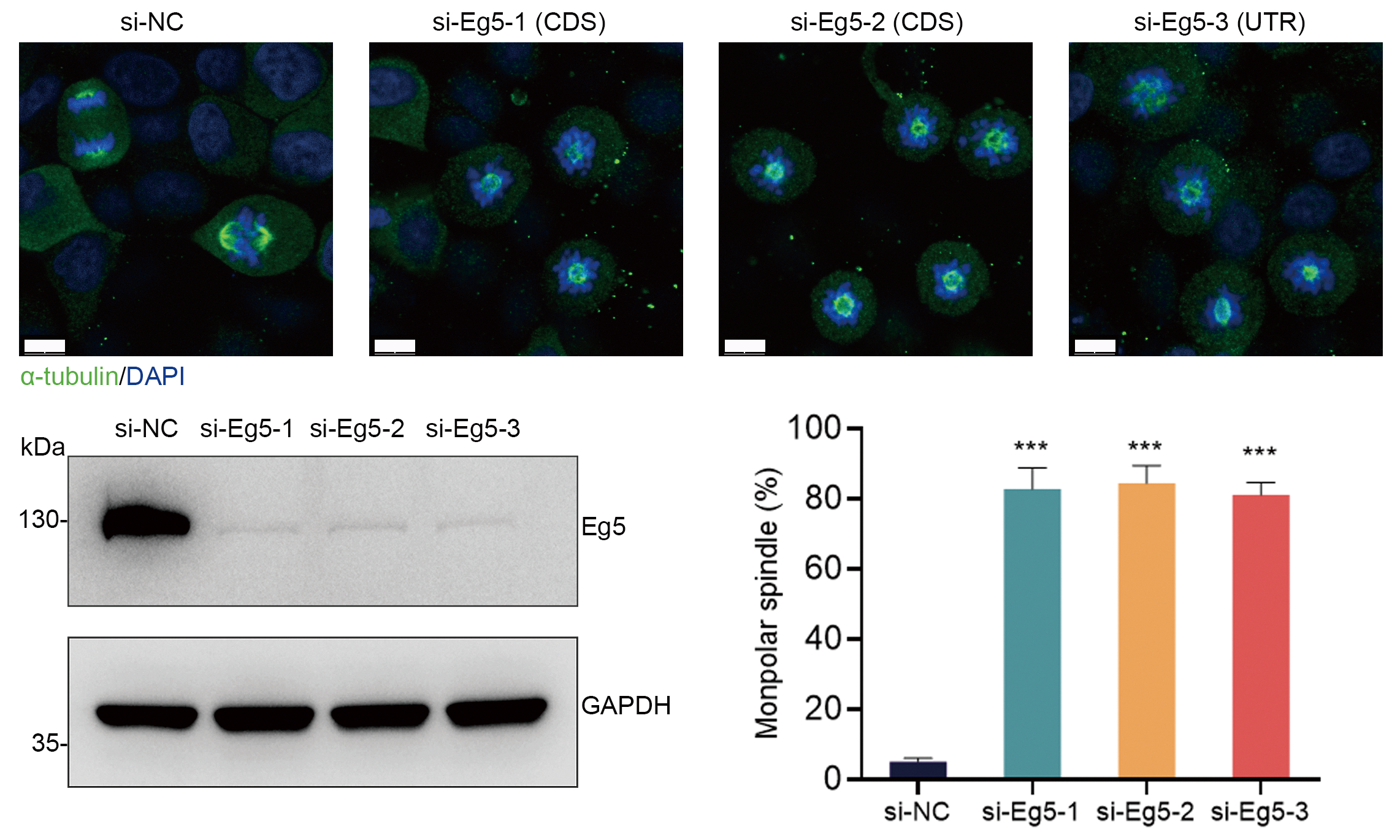
**FIGURE S3.** Eg5 knockdown caused monopole spindle. The phenotypes of mitotic spindle were determined by immunofluorescence staining using anti-α-tubulin and DAPI in HeLa cells. The Eg5 expression transfected with indicated siRNAs was determined by Western blot analysis. The percent of monopolar spindles were calculated. Scale bar, 10 μm. The mean ± SD from three independent experiments, with 75 cells per experimental group, is shown. The *P* values were determined by one-way ANOVA. ****P* < 0.001.
