# Supplementary material for: Eg5 UFMylation promotes spindle organization during mitosis
Source: Cell Death Dis. 2024 Jul 31;15(7):544. doi: 10.1038/s41419-024-06934-w (PMC11291904; doi:10.1038/s41419-024-06934-w)

FIGURE 2A

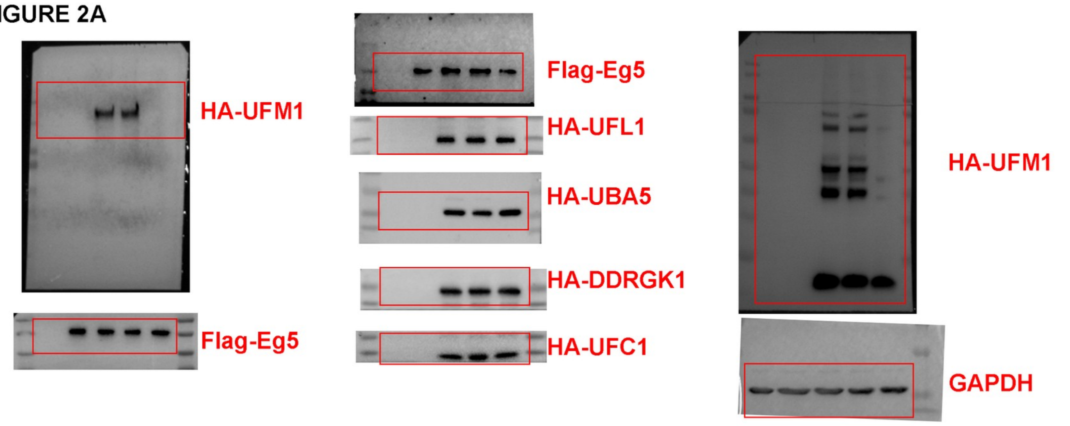

FIGURE 2B

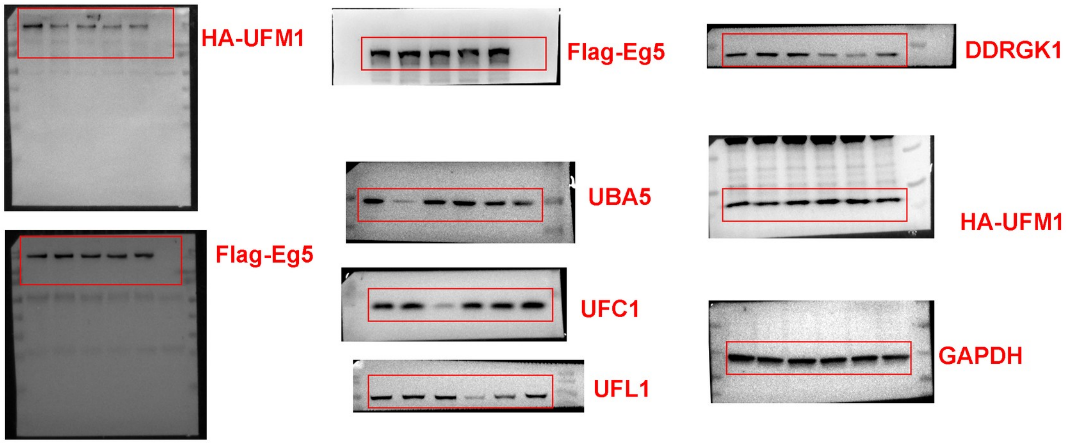

FIGURE 2C

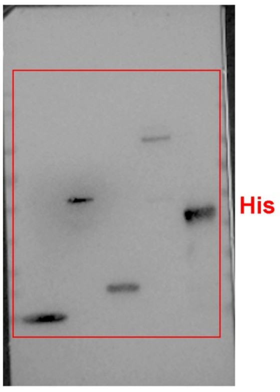

FIGURE 2D

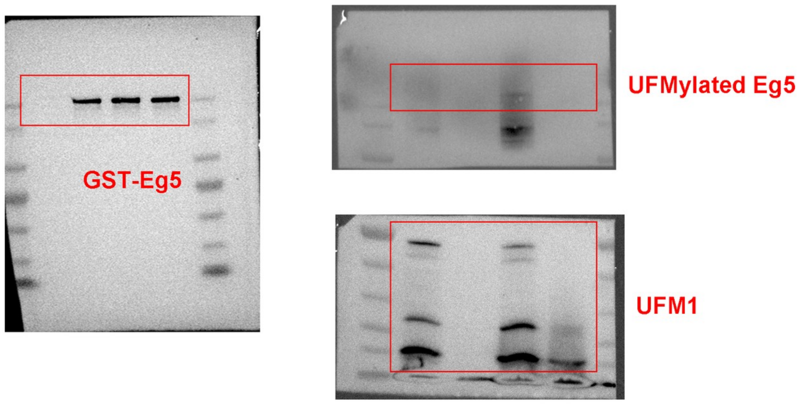

FIGURE 3A

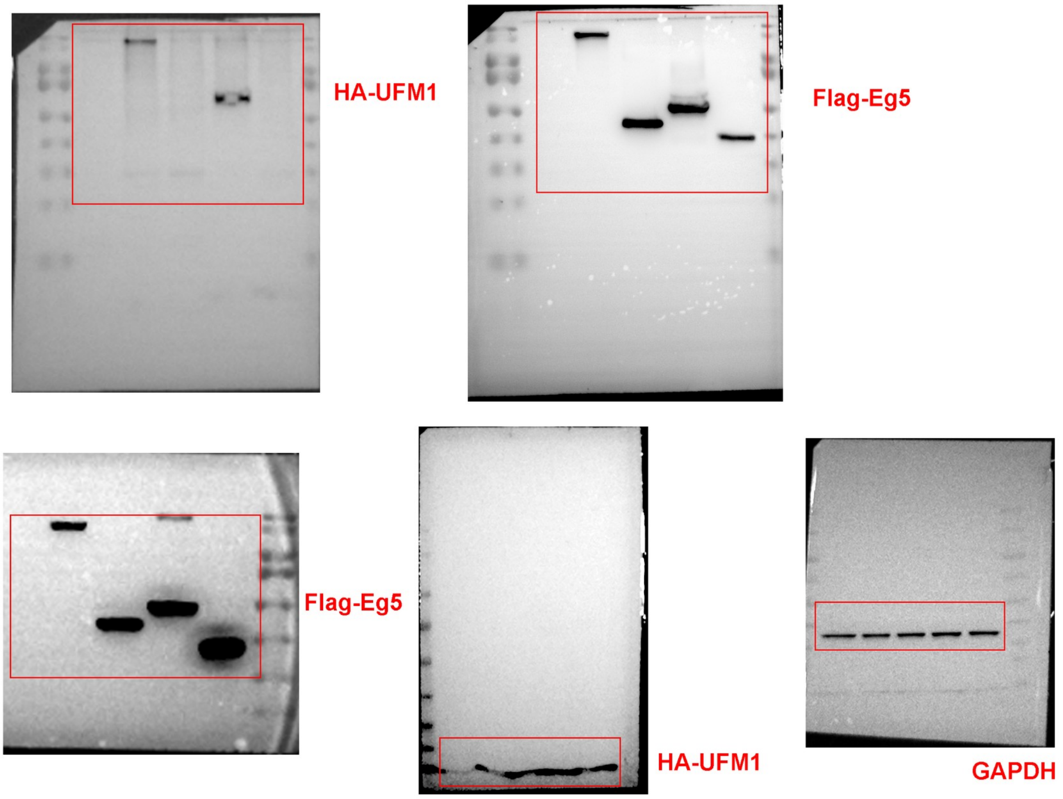

FIGURE 3B

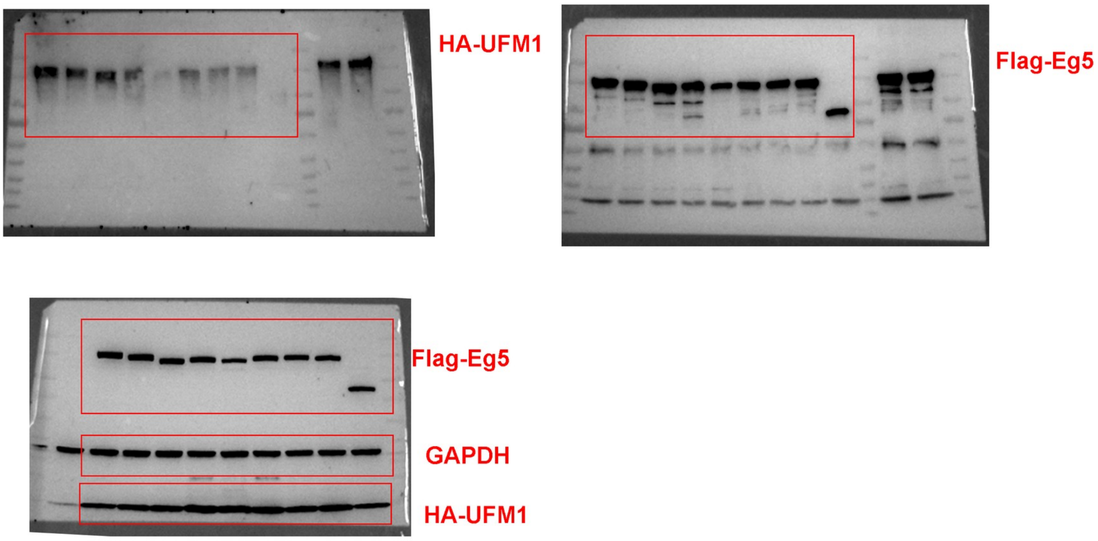

FIGURE 3C

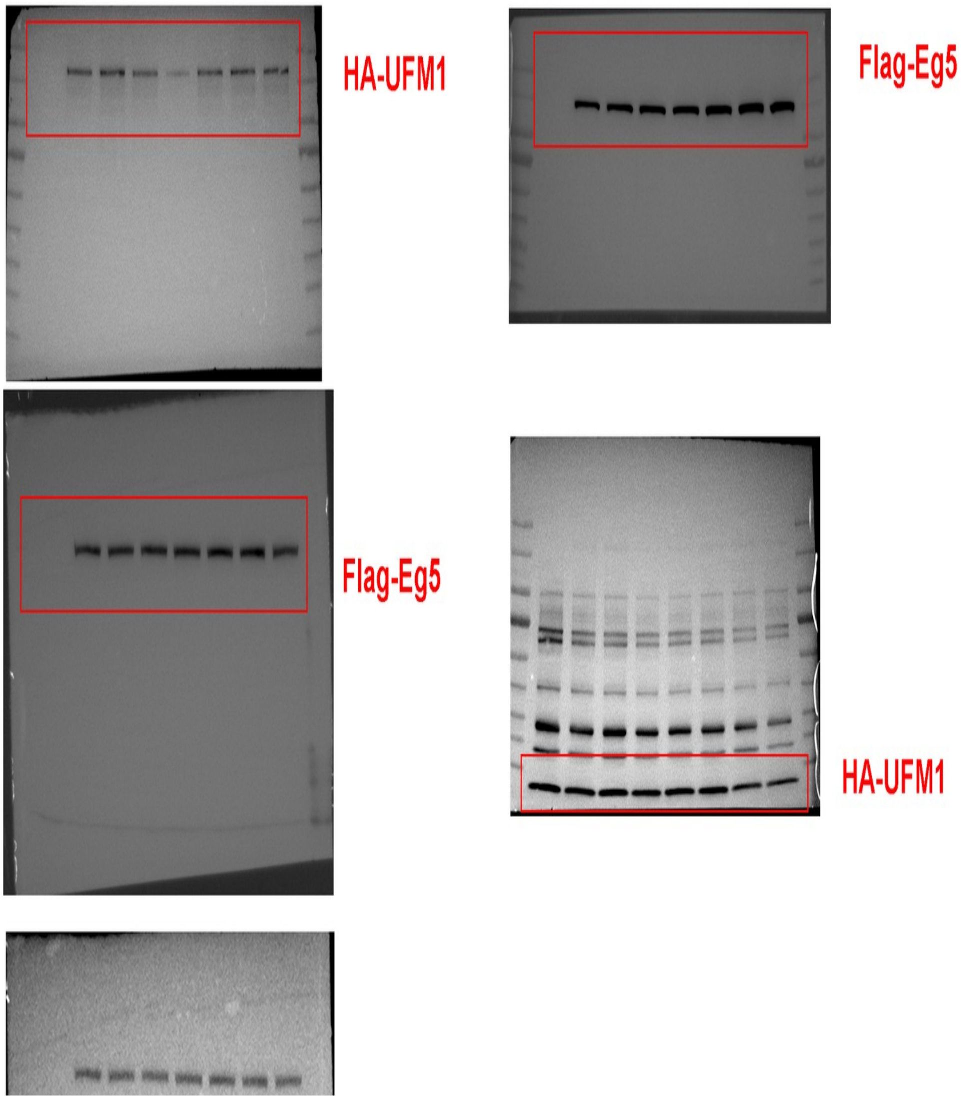

Supplement: Supplementary file 3 [file 41419_2024_6934_MOESM3_ESM.doc]
